# Diagnosis and mortality prediction of sepsis via lysophosphatidylcholine 16:0 measured by MALDI-TOF MS

**DOI:** 10.1038/s41598-020-70799-0

**Published:** 2020-08-14

**Authors:** Eun Hye Lee, Mi Hwa Shin, Jong-Min Park, Sang-Guk Lee, Nam Su Ku, Young Sam Kim, Moo Suk Park, Jae-Chul Pyun, Kyung Soo Chung

**Affiliations:** 1 Division of Pulmonology, Allergy and Critical Care Medicine, Department of Internal Medicine, Yongin Severance Hospital, Yonsei University College of Medicine, Yongin-si, Gyeonggi-do Republic of Korea; 2 Division of Pulmonary and Critical Care Medicine, Department of Internal Medicine, Institute of Chest Diseases, Severance Hospital, Yonsei University College of Medicine, 50-1 Yonsei-ro, Seodaemun-gu, Seoul, 03722 Republic of Korea; 3 Department of Materials Science and Engineering, Yonsei University, 50 Yonsei-ro, Seodaemun-gu, Seoul, Republic of Korea; 4 Department of Laboratory Medicine, Severance Hospital, Yonsei University College of Medicine, Seoul, Republic of Korea; 5 Department of Internal Medicine and AIDS Research Institute, Yonsei University College of Medicine, Seoul, Republic of Korea

**Keywords:** Phospholipids, Bacterial infection, Diagnostic markers, Predictive markers

## Abstract

Sepsis remains a critical problem with high mortality worldwide, but there is still a lack of reliable biomarkers. We aimed to evaluate the serum lysophosphatidylcholine (LPC) 16:0 as a biomarker of sepsis using matrix-assisted laser desorption/ionization time-of-flight mass spectrometry (MALDI-TOF MS). Patients admitted to intensive care unit at Severance Hospital from March 2017 through June 2018 were prospectively enrolled. The inclusion criteria were the fulfillment of at least two criteria of systemic inflammatory response syndrome (SIRS) or the presence of sepsis. Of the 127 patients, 14 had non-infectious SIRS, 41 had sepsis, and 72 had septic shock. The mean serum LPC 16:0 concentration (µmol/L) in non-infectious SIRS was significantly higher than in patients with sepsis and septic shock (101.1 vs. 48.92, *p* < 0.05; 101.1 vs. 25.88, *p* < 0.001, respectively). The area under the curve (AUC) predicting 28-day mortality using ΔLPC16:0 (D1-D0) levels was 0.7, which was comparable with the APACHE II score (AUC 0.692) and SOFA score (AUC 0.67). Mechanical ventilation, CRRT, lactate, **Δ** LPC16:0 (D1-D0) less than the cut-off value were significantly associated with 28-day mortality in multivariable analysis. Our results suggest that LPC16:0 could be a useful biomarker for sepsis diagnosis and mortality prediction in ICU patients.

## Introduction

Sepsis is a critical health problem and is one of the main causes of death in intensive care units (ICU) worldwide^1,2^. Although the sepsis mortality rate has decreased recently, it is still estimated to be between 35 and 55%^3^. As clinical information alone is rarely sufficient to detect sepsis, much effort has been expended to identify biomarkers for the early detection of sepsis, risk stratification, and prognostic prediction^4^. C-reactive protein (CRP) and procalcitonin (PCT) have been widely studied as sepsis biomarkers^5,6^, but it remains difficult to differentiate sepsis from other non-infectious inflammatory diseases. Recent evidence suggests that cell surface markers associated with immune dysfunction^7,8^ and a peripheral blood-based molecular assay, designated as “SeptiCyte LAB,” could be promising for discriminating sepsis from non-infectious systemic inflammation^9,10^. However, these approaches may require considerable time, effort, and cost. Since matrix-assisted laser desorption/ionization time-of-flight (MALDI-TOF) mass spectrometry (MS) was developed, it has been widely used for the analysis of peptides, whole proteins, and nucleotides. The advantages of MALDI-TOF MS compared to those of other analytical methods include easy sample preparation, sensitive detection (< fmol), wide detection range of up to 500 kDa, and short analysis time^11,12^.

In our previous study^12^, we identified differentially abundant low molecular weight molecules in the serum of healthy volunteers and sepsis patients using MALDI-TOF MS based on a parylene-matrix chip. The selected mass peaks of m/z (mass-to-charge) were 496.3 and 518.3 and tandem mass spectrometry was then performed to identify these molecules as LPC16:0. The aim of this present study was to confirm our previous identified LPC 16:0 associated with clinical outcome and evaluate its role as a biomarker for the diagnosis and mortality prediction of ICU sepsis patients.

## Methods

### Study design and cohort

Patients admitted to medical ICU (MICU) were prospectively enrolled from March 2017 through June 2018 at Severance Hospital, a 2,500-bed (30-bed medical ICU) university tertiary referral hospital in Seoul, South Korea. The study protocol involved two cohorts (Cohort 1: ER sepsis cohort, Cohort 2: MICU cohort). The inclusion criteria for the cohorts were an age > 19 years and fulfillment of at least two criteria of systemic inflammatory response syndrome (SIRS) or the presence of sepsis within the first 24 h after ICU admission. The definition of sepsis followed the revised sepsis 3 definition (infection + an increase ≥ 2 of a Sequential Organ Failure Assessment (SOFA) score)^2^. Septic shock was defined as sepsis with vasopressors required to maintain a mean arterial pressure of 65 mmHg or greater and serum lactate levels greater than 2 mmol/L in the absence of hypovolemia^2^. Patients with chronic liver disease, patients who refused consent, and patients whose blood specimens were lost were excluded from the study. All patients were observed for at least 28 days from the time of enrollment until death or discharge from the hospital.

**Figure 1:**
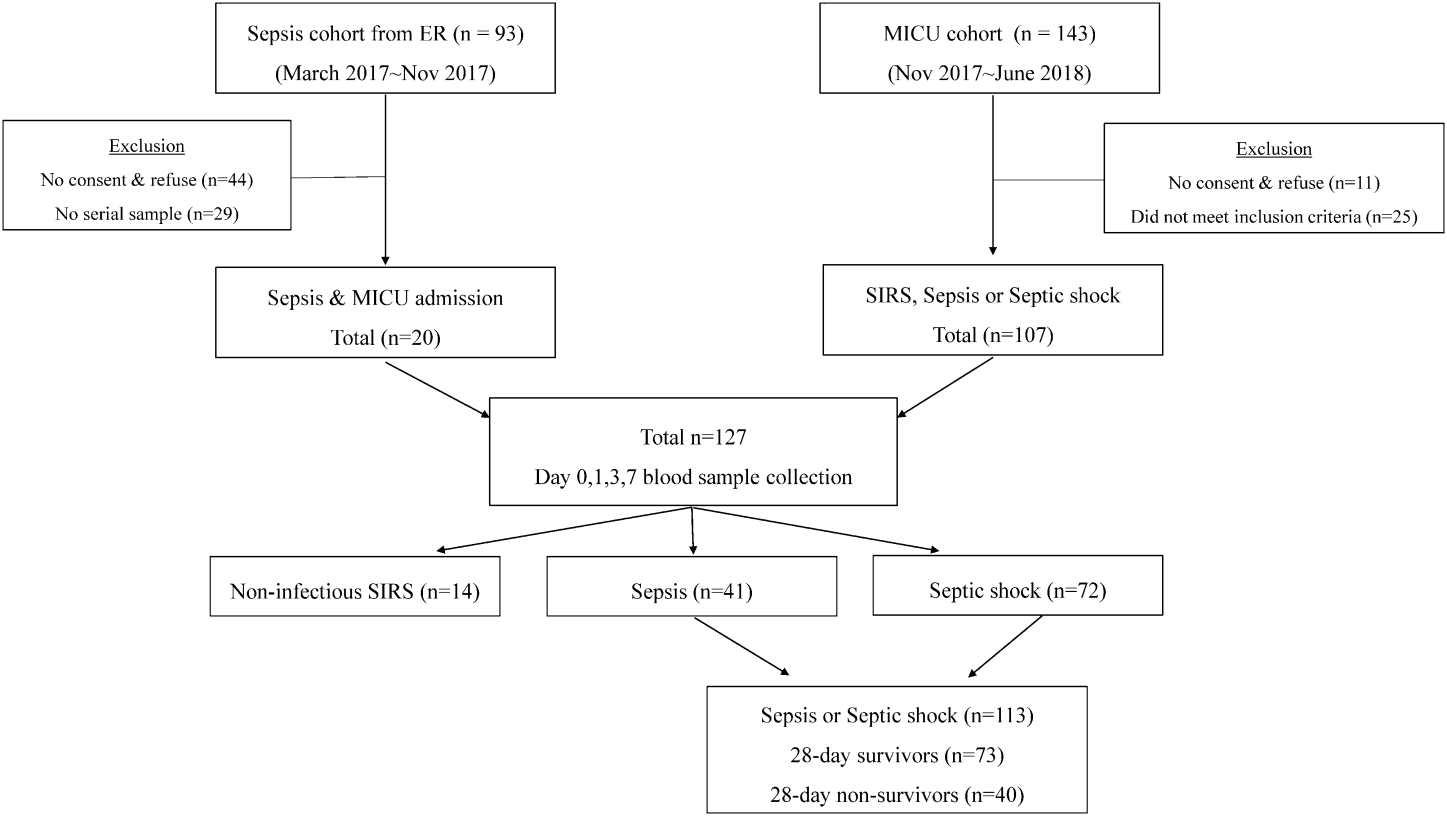
Study flowchart.

The study ultimately included a total of 127 patients between cohort 1 and 2. Among these, 14 patients were classified as non-infections SIRS, 41 as sepsis, and 72 as septic shock, according to the sepsis 3 definition. All patients were treated with standard therapy for sepsis and septic shock, according to international guidelines^13^. Patients were classified as either survivors or non-survivors according to the 28-day post-enrollment outcome (Fig. 1).

### Data collection

Clinical and laboratory patient data were collected from the hospital medical records. The following clinical data were collected: age, gender, Charlson Comorbidity index (CCI)^14^, source of infection, microbiological blood culture results, and 28-day outcome. The Acute Physiology and Chronic Health Evaluation II (APACHE II) and SOFA scoring systems were used to determine the severity of patient condition over the first 24 h following admission (D0). Information regarding inflammatory markers, including white blood cell (WBC) count, C-reactive protein (CRP), and procalcitonin (PCT), was also collected from medical records.

### Blood sample collection and measurement of LPC16:0

Whole blood was collected on the day of ICU admission (D0) and on day 1 (D1), day 3 (D3), and day 7 (D7) after admission. First, whole blood samples were vortexed for 30 s, and centrifuged at 12,700 × *g* for 5 min. For each sample, the pellet was discarded, and 100 μL of serum was added to 400 μL of acetonitrile and vortexed for 30 s. The mixture was centrifuged at 12,700 × *g* for 10 min. Finally, 300 μL of the supernatant was analyzed using a MALDI-TOF MS. An equal volume of 0.1% trifluoroacetic acid aqueous solution was added to each serum extract prior to MALDI-TOF MS analysis. Pure LPC16:0 solution was prepared for mass comparison and quantitative MALDI-TOF MS. For measurement of LPC16:0 in patient serum, a MALDI-TOF MS with a Parylene matrix chip was used to measure small molecules at m/z ratios of less than 500 Da^15^. The LPC16:0 standard and serum extracts were dispensed onto Parylene matrix chip arrays (0.5 μL of sample per spot) and quantified using a Microflex mass spectrometer (Bruker Daltonics; Bremen, Germany) equipped with a nitrogen laser (337 nm). Detailed methods for LPC measurement were provided in our previous study^12^. Quantitative analysis of the LPC16:0 standard on a Parylene matrix chip confirmed the linear relationship between the intensity and concentration for LPC16:0 (Supplementary Figure S1). For the validation of the identity of the LPC16:0 peak in the clinical serum samples, MS/MS analysis was carried out using a pure LPC16:0 standard solution. Measurement of LPC16:0 was performed on D0, D1, D3, and D7 serum samples for each patient. All clinicians and patients participating in the study were blinded to the LPC results.

### Statistical analysis

Continuous variables are presented as mean ± standard deviation (SD) or median and interquartile range (IQR). Continuous variables were compared using Student’s t-test or the Mann–Whitney U test. Categorical variables were reported as a number and percentage and were compared using the chi-square test or Fisher’s exact test. A non-parametric Kruskal–Wallis test was used to compare three or more groups for qualitative parameters (followed by post-hoc analysis). The correlations between LPC levels and other variables were determined using the Spearman correlation test. Multiple Cox-regression analysis was used to determine whether LPC levels were independently associated with 28-day mortality outcomes. The Kaplan–Meier method was used to generate survival curves that were analyzed using the log-rank test. In all tests, a p-value < 0.05 was considered significant. SPSS v23.0 (IBM, Armonk, NY, USA) was used for statistical analysis and GraphPad Prism 7 (Graph-Pad, San Diego, CA, USA) was used for graphical representations. ROC curves were generated and AUC analysis was performed using the MedCalc software (version 16.4.3; MedCalc, Oostende, Belgium).

### Ethics

The research protocol was approved by the Institutional Review Board (IRB) of Severance Hospital (ER cohort IRB number: 4-2016-0605, MICU cohort IRB number: 4-2017-0654). Written informed consent was obtained from patients or their guardians. All methods were carried out in accordance with relevant guidelines and regulations.

## Results

### Baseline characteristics of study patients

A total of 127 patients were enrolled in the present study. Among these, 14 fulfilled at least 2 SIRS criteria that suggest non-infectious SIRS, 41 were classified as having sepsis and 72 were classified as having septic shock. Of the 113 patients who met the sepsis diagnostic criteria, 73 survived and 40 had died at 28 days after ICU admission (Fig. 1). The clinical and laboratory characteristics of sepsis and septic shock patients admitted to the ICU are shown in Table 1. We compared the clinical and laboratory features of 28-day survivors and non-survivors. The median age was 70 overall, and more participants were male than female (70/113, 61.9%). The most common site of infection was pulmonary in both groups. Urinary tract infections were more common in survivors (19.2% vs. 2.5%, *p* = 0.018), while the proportion of abdominal infections was significantly higher in non-survivors (11.0% vs. 32.5%, *p* = 0.006). The proportion of septic shock and bacteremia did not differ significantly between survivors and non-survivors, but mechanical ventilation (46.6% vs. 77.5%, *p* = 0.003) and continuous renal replacement therapy (CRRT) (27.4% vs. 52.5%, *p* = 0.002) were more frequently performed in non-survivors. The SOFA (8 [IQR, 6–10] vs. 9 [IQR, 8–13], *p* = 0.003) and APACHE II scores (23 [IQR, 16–32] vs. 31 [IQR, 23–35], *p* = 0.001), as well as serum lactate levels (2.1 [IQR, 1.4–3.3] vs. 3.7 [IQR, 1.7–8.9]; *p* = 0.002) obtained in the initial laboratory test were lower for survivors compared to those for non-survivors. Baseline characteristics of 14 non-infectious SIRS patients were shown in Supplementary Table S1.

**Table 1:** Comparison of characteristics of sepsis and septic shock patients according to 28-day mortality.

	All patients (N = 113)	Survivors (N = 73)	Non-survivors (N = 40)	*P*-value
**Variables**				
Age (years), median, [IQR]	70 [60.5–77.5]	70 [58.8–77.2]	69 [61–78]	0.989
Gender, male, N (%)	70 (61.9)	44 (60.3)	26 (65.0)	0.479
BMI (kg/m^2^)	21.8 [19.2–25.1]	22.3 [18.8–25.3]	21.4 [19.3–24.4]	0.501
**Site of infection, N (%)**				
Pulmonary	65 (57.5)	43 (58.9)	22 (55.0)	0.676
Urinary tract	15 (13.3)	14 (19.2)	1 (2.5)	0.018
Abdomen^a^	21 (18.6)	8 (11.0)	13 (32.5)	0.006
Skin and soft tissue	5 (4.4)	3 (4.1)	2 (5.0)	1.0*
Others^b^	7 (6.2)	5 (6.8)	2 (5.0)	1.0*
Charlson comorbidity index	5 (4–7)	5 (4–6)	5 (4–8)	0.061
**Major comorbidities**				
Malignancy	29 (25.7)	16 (21.9)	13 (32.5)	0.079
DM	46 (40.7)	28 (38.4)	18 (45.0)	0.158
CKD or ESRD	30 (26.5)	18 (24.7)	12 (30.0)	0.262
CHF	13 (11.5)	7 (9.6)	6 (15.0)	0.339*
**Clinical parameters**				
Sepsis	41 (36.3)	29 (39.7)	12 (30.0)	0.149
Septic shock	72 (63.7)	44 (60.3)	28 (70.0)	0.149
Bacteremia	36 (31.9)	23 (31.5)	13 (32.5)	0.663
Mechanical ventilation	65 (57.5)	34 (46.6)	31 (77.5)	0.003
CRRT	41 (36.3)	20 (27.4)	21 (52.5)	0.002
**Clinical severity score, D0**				
APACHE II score	26 [19–32.5]	23 [16–32]	31 [23–35]	0.001
SOFA score	8 [7–11]	8 [6–10]	9 [8–13]	0.003
**Laboratory parameters, D0**				
Leukocytes (× 10^6^/mL)	13.7 [7.1–19.2]	13.2 [7.7–19.0)	14.1 [6.1–19.5]	0.783
Platelets (× 10^6^/mL)	144 [78.5–239]	160 [87.3–239.0]	132 [45–239]	0.305
CRP (mg/L)	171.7 [89.7–281.2]	212.2 [116.8–288.6]	158.9 [72.5–217.9]	0.034
Procalcitonin (ng/mL)	4.1 [1.1–26.6]	3.7 [0.9–29.6]	5.6 [1.7–21.4]	0.755
Lactate (mmol/L)	2.3 [1.6–4.6]	2.1 [1.5–3.2]	3 [1.9–8.9]	0.002
Albumin (g/dL)	2.5 [2.1–2.8]	2.6 [2.1–2.8]	2.4 [2.1–2.8]	0.072
Total Bilirubin (mg/dL)	0.6 [0.3–1.2]	0.6 [0.3–0.9]	0.7 [0.4–2.0]	0.079
BUN (mg/dL)	32.8 [20.0–50.8]	30.4 [19.9–49.3]	34.8 [23.4–52.6]	0.329
Creatinine (mg/dL)	1.7 [0.8–2.8]	1.4 [0.7–2.8]	2.1 [0.9–3.0]	0.19

*BMI* body mass index, *DM* diabetes mellitus, *CKD* chronic kidney disease, *ESRD* end stage renal disease, *CHF* congestive heart failure, *CRRT* continuous renal replacement therapy, *APACHE* Acute Physiology and Chronic Health Evaluation, *SOFA* Sequential Organ Failure Assessment, *CRP* C-reactive protein, *BUN* Blood urea nitrogen, *ABGA* arterial blood gas analysis.

Values are expressed as n (%) or median [interquartile range] unless otherwise indicated.

*Fisher exact test.

^a^Abdomen: gastrointestinal and hepatobiliary infection, peritonitis.

^b^Others: meningitis, spinal abscess, septic arthritis, primary unknown infection.

### LPC16:0 (D0) concentration in patients with non-infectious SIRS, sepsis, and septic shock

**Figure 2:**
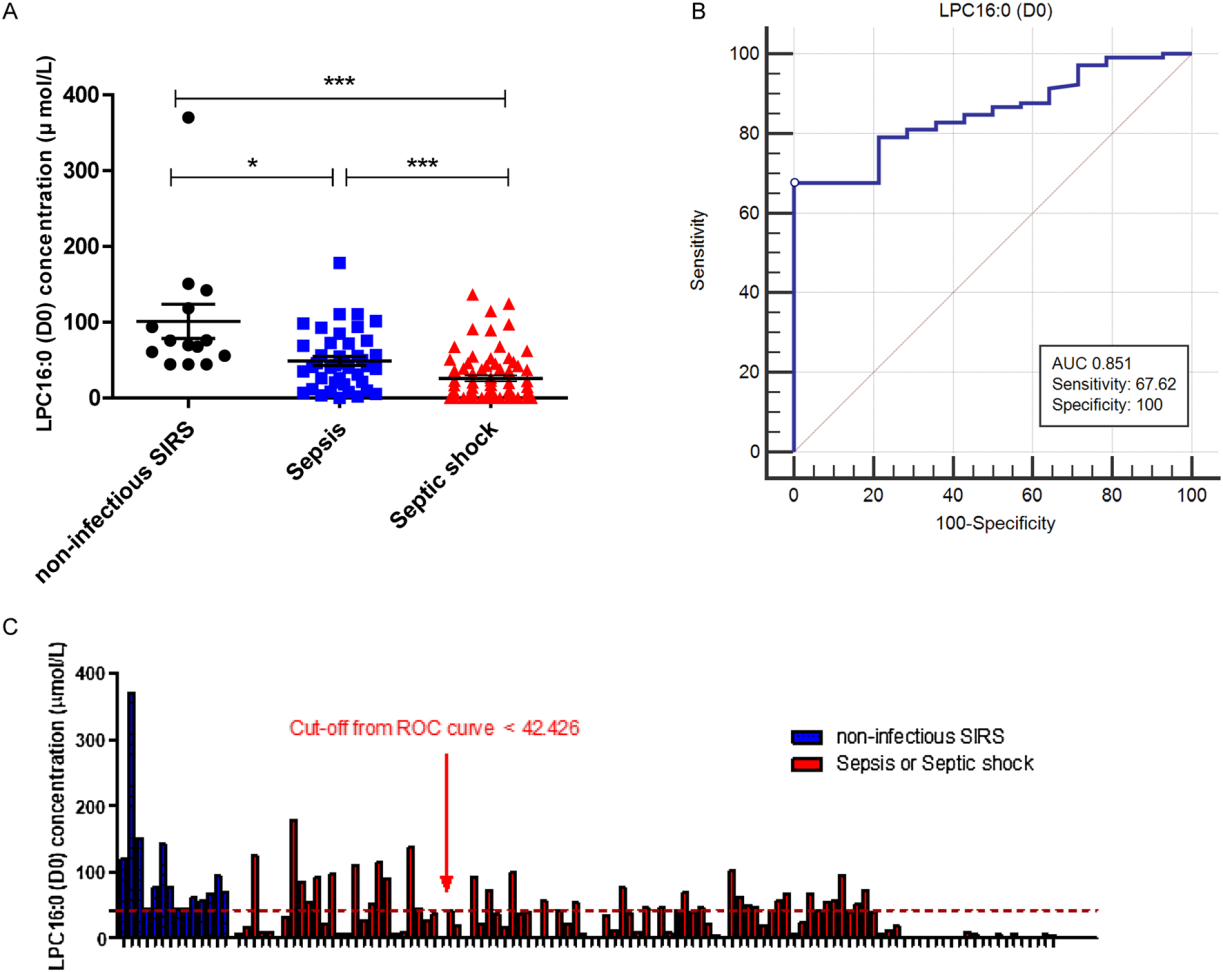
(**A**) LPC 16:0 (D0) concentration in patients with non-infectious SIRS (n = 14), Sepsis (n = 41) and Septic shock (n = 72). (**B**) ROC curve of LPC16:0 (D0) for diagnosis of sepsis. (**C**) Analysis results of the LPC16:0 (D0) concentration (µmol/L) from non-infectious SIRS (n = 14), sepsis and septic shock patients (n = 113). Data provided are the mean ± SEM, *P < 0.05, **P < 0.01 ***P < 0.001, analyzed by one-way ANOVA with Bonferroni’s post hoc test.

The baseline LPC16:0 concentration (µmol/L) at ICU admission (D0) is shown in Fig. 2. The mean serum LPC concentration in septic shock patients was significantly lower than in that in those with non-infectious SIRS (25.88 vs*.* 101.1, *p* < 0.001) and sepsis (25.88 vs*.* 48.92, *p* < 0.001). The mean serum LPC concentration in sepsis patients was also significantly lower than that in non-infectious SIRS patients (48.92 vs*.* 101.1, *p* < 0.05). Figure 2B shows ROC curve of LPC16:0 (D0) for diagnosis of sepsis. The AUC was 0.851 with sensitivity 67.62% and specificity 100% using LPC16:0 cut off value < 42.426 µmol/L.

### Comparison of LPC16:0 concentration between 28-day survivors and non-survivors among sepsis and septic shock patients

**Figure 3:**
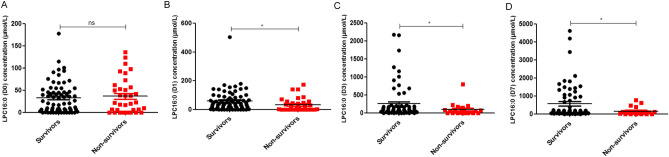
Comparison of LPC16:0 (D0–D7) concentration between survivors and non-survivors in sepsis patients (number of survivors and non-survivors; D0 73:40, D1 73:37, D3 73:28, D7 73:23, respectively). Data provided are the mean ± SEM, *P < 0.05, **P < 0.01, ***P < 0.001 analyzed by Student’s unpaired two-tailed t test.

**Figure 4:**
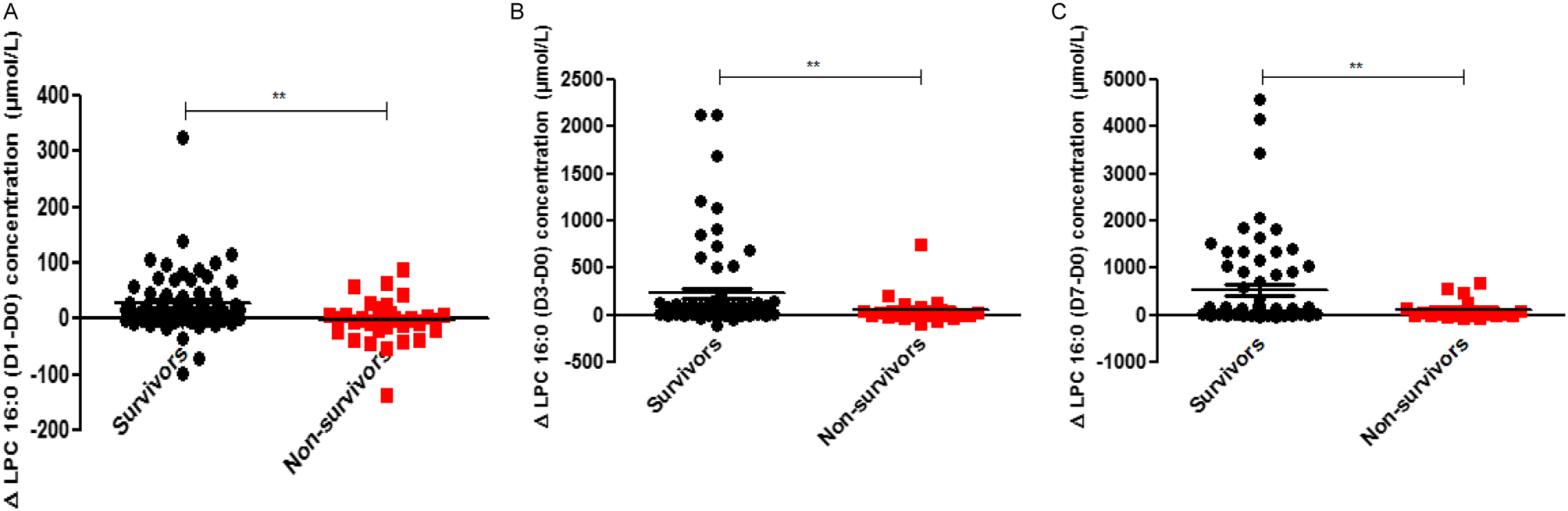
Comparison of Δ LPC16:0 concentration (D1–D0, D3–D0, D7–D0) between survivors and non-survivors in sepsis patients (number of survivors and non-survivors; D0 73:40, D1 73:37, D3 73:28, D7 73:23, respectively), Data provided are the mean ± SEM, *P < 0.05, **P < 0.01, ***P < 0.001 analyzed by Student’s unpaired two-tailed t test.

The concentration of LPC16:0 measured on D0, D1, D3, and D7 in both survivors (n = 73) and non-survivors (n = 40) was determined using MALDI-TOF MS (Fig. 3). There was no significant difference in LPC16:0 concentration at ICU admission (D0) between the two groups. On D1, D3, and D7, the concentration of LPC was lower in non-survivors than in survivors (p < 0.05) (Fig. 4). The survivor group showed a statistically significant (P < 0.01) increase in the amount of LPC16:0 over time compared to the non-survivor group.

### Association between changes in serum LPC16:0 levels and 28-day mortality

**Figure 5:**
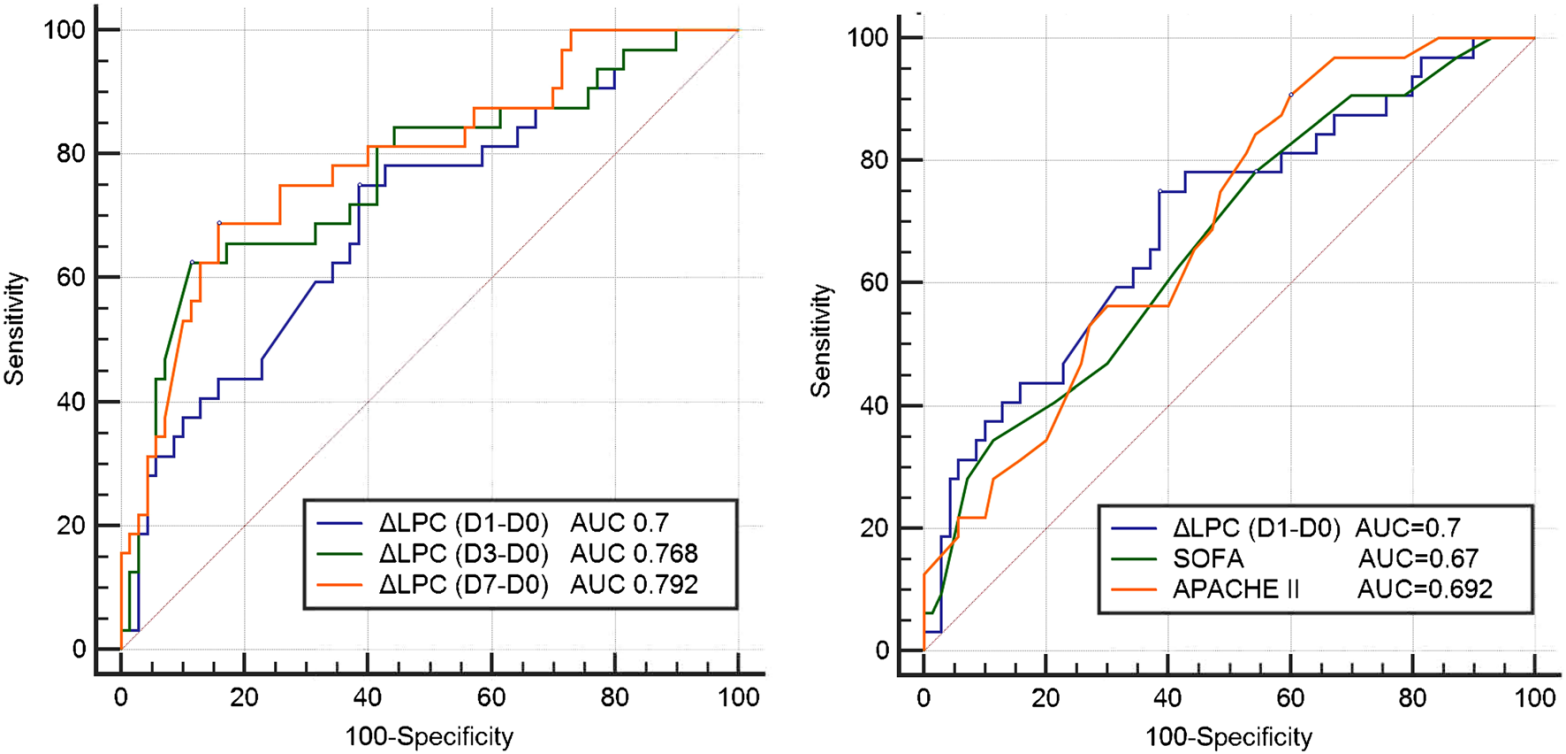
(**A**) ROC curve of Δ LPC (D1–D0, D3–D0, D7–D0) for predicting 28-day mortality (**B**) ROC curve of Δ LPC (D1–D0) compared to SOFA, APACHE II score. In ROC curve comparisons, the AUC of ΔLPC (D1–D0) was higher than SOFA (AUC 0.7 vs. 0.67, *p* = 0.712) or APACHE II score (AUC 0.7 vs. 0.692, *p* = 0.916) for predicting 28-day mortality, although there is no statistical difference.

**Figure 6:**
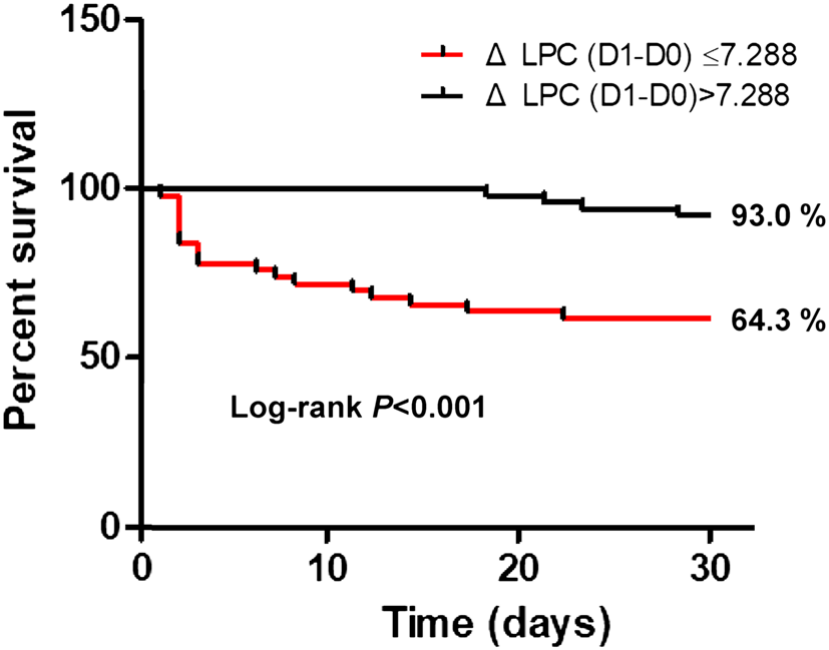
Kaplan–Meier survival analysis showed that the 28-day mortality of patients with plasma ΔLPC (D1–D0) > 7.288 was higher than that of patients with ΔLPC (D1–D0) < 7.288 (*p* < 0.001).

Receiver operating characteristic (ROC) curves were generated to compare changes in LPC16:0 concentration and clinical severity index for prediction of 28-day mortality (Fig. 5). The area under the curve (AUC) for ΔLPC16:0 (D1-D0) was 0.7 (95% confidence interval [CI] 0.602–0.787), while the AUCs for the APACHE II score and SOFA score were 0.692 (95% CI 0.593–0.780) and 0.67 (95% CI 0.570–0.766), respectively. According to this comparison, the AUC of ΔLPC16:0 (D1-D0) is higher than that of the APACHE II score (AUC 0.7 vs. 0.692, p = 0.916) and SOFA score (AUC 0.7 vs. 0.67, *p* = 0.712), although this difference is not statistically significant. Patients were divided into two groups according to this cut-off value and subjected to a cox proportional hazard model analysis involving several demographic and clinical characteristics (Table 2). According to univariate analysis, the Charlson Comorbidity Index (CCI), abdominal origin of sepsis, mechanical ventilation, application of CRRT, lactate level, and ΔLPC D1 were associated with 28-day mortality. According to multivariate analysis, mechanical ventilation (HR 12.415; 95% CI 2.113–72.947), CRRT (HR 4.236; 95% CI 1.029–17.435), lactate (D0) (HR 1.455; 95% CI 1.035–2.045), ΔLPC D1 less than the cut-off value (**Δ** LPC16:0 (D1–D0) ≤ 7.288; HR 6.491; 95% CI 1.614–26.100) were significantly associated with an increased 28-day mortality. Figure 6 shows the Kaplan–Meier survival analysis of the patient groups classified according to the ΔLPC (D1–D0) cut-off, and these 28-day survival rates differed significantly (*P* < 0.001).

**Table 2:** Cox proportional hazard model of 28-day mortality.

Variable	Univariable analysis			Multivariable analysis		
	HR	95% CI	*P*-value	Adjusted HR	95% CI	*P*-value
Age (year)	1.001	0.974–1.028	0.967			
Sex (male)	1.356	0.583–3.155	0.480			
BMI (kg/m^2^)	1.008	0.928–1.095	0.843			
Charlson comorbidity index	1.196	1.010–1.417	0.038	1.269	0.958–1.681	0.097
**Site of infection**						
Pulmonary	1			1		
Urinary tract	0.154	0.019–1.259	0.081	0.213	0.014–3.291	0.268
Abdomen^a^	3.391	1.137–10.112	0.028	9.074	1.443–57.073	0.019
Skin and soft tissue	1.439	0.222–9.335	0.703	10.469	0.680–161.090	0.092
Others^b^	0.863	0.153–4.858	0.867	0.001	0.000–5.119	0.999
Septic shock	1.926	0.786–4.719	0.152			
Mechanical ventilation	3.784	1.511–9.476	0.003	12.415	2.113–72.947	0.005
CRRT	3.750	1.599–8.793	0.002	4.236	1.029–17.435	0.046
Bacteremia	1.208	0.517–2.819	0.663			
Lactate	1.388	1.163–1.658	< 0.001	1.455	1.035–2.045	0.031
Procalcitonin	0.993	0.980–1.007	0.347			
**Δ** LPC16:0 (D1–D0) ≤ 7.288	4.778	1.878–12.155	< 0.001	6.491	1.614–26.100	0.008

*HR* hazard ratio, *CI* confidence interval, *BMI* body mass index, *CRRT* continuous renal replacement therapy, *SOFA* Sequential Organ Failure Assessment, *APACHE* Acute Physiology and Chronic Health Evaluation.

^a^Abdomen: gastrointestinal, hepatobiliary infection, peritonitis.

^b^Others: meningitis, spinal abscess, septic arthritis, primary unknown infection.

## Discussion

In the present study, we found that the changes in LPC16:0 concentration over time were closely associated with 28-day mortality in sepsis patients under intensive care. Patients with persistently low LPC16:0 have shown an increased risk of death whereas those who have recovered LPC16:0 had better prognosis.

Previous studies have found that bioactive lipids have significant effects on immune and inflammatory cells, and are regarded as mediators during the development of sepsis^16–19^. In septic condition, activation of hepatic *de nove* lipogenesis lead to elevation of phosphatidylcholine (PC), triglycerides (TGs), free fatty acid^16,17^. LPC is produced by the action of phospholipase A2 (PLA2) on PC and can be transported back to the liver and converted to PC by lysophosphatidylcholine acyltransferase (LPCAT)^20^. LPC has been known to play a role in the immune regulation and stimulation of immune cells, including monocytes, macrophages, T lymphocytes, and neutrophils^21–23^. Several studies have reported that the serum concentrations of LPC subtypes 16:0, 18:0, 18:1, and 18:2, as well as total LPC were lower in sepsis patients compared to those in healthy controls^21,24^ and another recent study suggested that LPC16.0 predicts 28 day and 90-day mortality better than other LPC subtypes^25^. Impaired metabolic homeostasis is thought to be the cause of sustained low levels of LPC in sepsis patients^20^.

In our previous study, we identified LPC16:0 as a useful biomarker for diagnosis of sepsis via novel technique using MALDI-TOF MS based on a parylene-matrix chip^12^. The sensitivity and selectivity of LPC16:0 to sepsis diagnosis was estimated to be 97.9% and 95.5%, respectively by using MALDI-TOF MS^12^. However, our previous study had limitation in comparing healthy control and sepsis patients. Many previous studies have been conducted to distinguish between sepsis and SIRS^9^, which continues to be a difficult challenge^26^. Furthermore, biomarkers that not only diagnose sepsis but also predict prognosis are more difficult to find. In this current study, the significant difference in LPC16:0 levels in patients with non-infectious SIRS and sepsis on day0 suggested that LPC16:0 could be a useful diagnostic biomarker of sepsis (101.1 vs. 48.92, *p* < 0.05) (Fig. 2). We also found that the change in LPC levels over time with sepsis was an important prognostic tool for predicting mortality in ICU. The AUC value for the 28-day mortality prediction of the ΔLPC (D1-D0) value was comparable to those for the APACHE II and SOFA scores, which are well-known severity indexes in ICU patients [ΔLPC (D1–D0) AUC = 0.7, APACHE II AUC = 0.692, SOFA AUC = 0.670]. Our study also found that ΔLPC (D3–D0) and ΔLPC (D7–D0) had predictive value for 28-day mortality [ΔLPC (D3–D0) AUC = 0.768, ΔLPC (D7–D0) AUC = 0.792]. According to these results, if the LPC16:0 concentration does not increase beyond a cut-off during the course of treatment for 7 days after the diagnosis of sepsis, the risk of mortality significantly increases. The differences in mortality risk predicted by changes in LPC concentration were also significant at initial 24 h after sepsis diagnosis (Fig. 6). Considering the dynamic clinical course of sepsis, which occurs over a short period of time, this finding is clinically significant because measuring changes in LPC concentration between D1 and D3 compared to D0 is as effective as D7 for predicting mortality. Park DW et al*.* also reported that changes in LPC levels were associated with mortality; however, they used changes in LPC concentration in combination with those in other clinical markers, such as procalcitonin, for predicting patient mortality^27^.

In the present study, we found no significant correlation between LPC levels or changes in LPC levels and the established clinical severity indexes APACHE II and SOFA. This apparent lack of correlation might be due to the biological properties of LPC as an immunoregulatory ligand for cells of the innate and adaptive immune systems^22,28^. Previous studies have reported that LPC treatment in an experimental animal sepsis model decreased the level of pro-inflammatory cytokines^23,29^, and, in the present study, we observed that the LPC level was inversely correlated with the levels of the inflammatory markers procalcitonin and lactate (Supplementary Figure S2). Clinical severity indexes, such as APACHE II and SOFA scores could have limited value in classifying the immunologic and inflammatory status of sepsis, which is representative of organ failure. Therefore, monitoring LPC recovery together with clinical indices may be the best way to examine the course of disease for predicting the prognosis of sepsis.

The present study has several limitations. It was conducted at a single center and only included sepsis patients admitted to the ICU; it may, thus, have associated bias. Other inflammatory cytokines that might be related to changes in LPC levels were not monitored. The value of LPC16:0 for diagnosing sepsis were not sufficient due to small number and high severity of the control group and the absolute value of LPC 16:0 at initial day was not able to discriminate survivors and non-survivors. Despite these limitations, our study highlights the power of MLLDI-TOF MS of analytes on a Parylene matrix chip^15,30^ for facilitating the analysis of subtypes of LPC accurately and without interference from organic matrix mass peaks. By performing the serial analysis of patient samples over the course of sepsis, we were able to understand the association between changes in LPC levels and disease prognosis and mortality, which can enable the better classification of patients in early stages of sepsis. A large, multicenter population study, however, is needed to verify these results in sepsis patients.

## Conclusions

In the present study, we used MALDI TOF MS to measure the levels of serum LPC16:0 in sepsis patients requiring ICU care and found that changes in the concentration of LPC16:0 had predictive potential for determining the prognosis and mortality. Sustained low levels of LPC were associated with poor prognosis and high mortality risk, and these patients are, therefore, candidates for intensive care and a more rigorous treatment plan.

## Supplementary information

Supplementary file1.

## Data Availability

All datasets generated and analysed during this study are available from the corresponding author on reasonable request.
