# Supplementary material for: Diagnosis and mortality prediction of sepsis via lysophosphatidylcholine 16:0 measured by MALDI-TOF MS
Source: Sci Rep. 2020 Aug 14;10:13833. doi: 10.1038/s41598-020-70799-0 (PMC7427783; doi:10.1038/s41598-020-70799-0)
Supplement: Supplementary file 1 — Supplementary file1. [file 41598_2020_70799_MOESM1_ESM.docx]

**Diagnosis and mortality prediction of sepsis via lysophosphatidylcholine 16:0 measured by MALDI-TOF MS**

Eun Hye Lee^1^, Mi Hwa Shin^2^, Jong-Min Park^3^, Sang-Guk Lee^4^, Nam Su Ku^5^, Young Sam Kim^2^, Moo Suk Park^2^, Jae-Chul Pyun^3^* and Kyung Soo Chung^2^*

**Supplementary Table S1. Baseline characteristics of 14 non-infectious SIRS patients**

| **Variables** | **Non-infectious SIRS** |
| --- | --- |
|  | **(N=14)** |
| Age, years, median, [IQR] | 60 [48.3-76.8] |
| Gender, male, N (%) | 8 (57.1) |
| BMI, kg/m^2^ | 20.4 [17.6-25.3] |
| **Cause of ICU admission**, N (%) |  |
| Heart failure | 4 |
| Gastrointestinal bleeding | 3 |
| Hemoptysis | 2 |
| Mental change | 1 |
| Others* | 4 |
| **Charlson comorbidity index** | 5.5 (2.75-6) |
| **Major comorbidities** |  |
| Malignancy | 4 (28.6) |
| DM | 5 (35.7) |
| CKD or ESRD | 4 (28.6) |
| CHF | 4 (28.6) |
| **Clinical parameters** |  |
| Mechanical ventilation | 10 (71.4) |
| CRRT | 4 (28.6) |
| **Clinical severity score, D0** | |
| APACHE II score | 21 [13.8-32.0] |
| SOFA score | 5 [3-8] |
| **28-day mortality, N (%)** | 3 (21.4) |

*2 liver failure, 1 renal failure, 1 asphyxia

Supplementary Figure S1. Graph of LPC16:0 intensity (a.u) vs. LPC16:0 concentration (μmol/L)


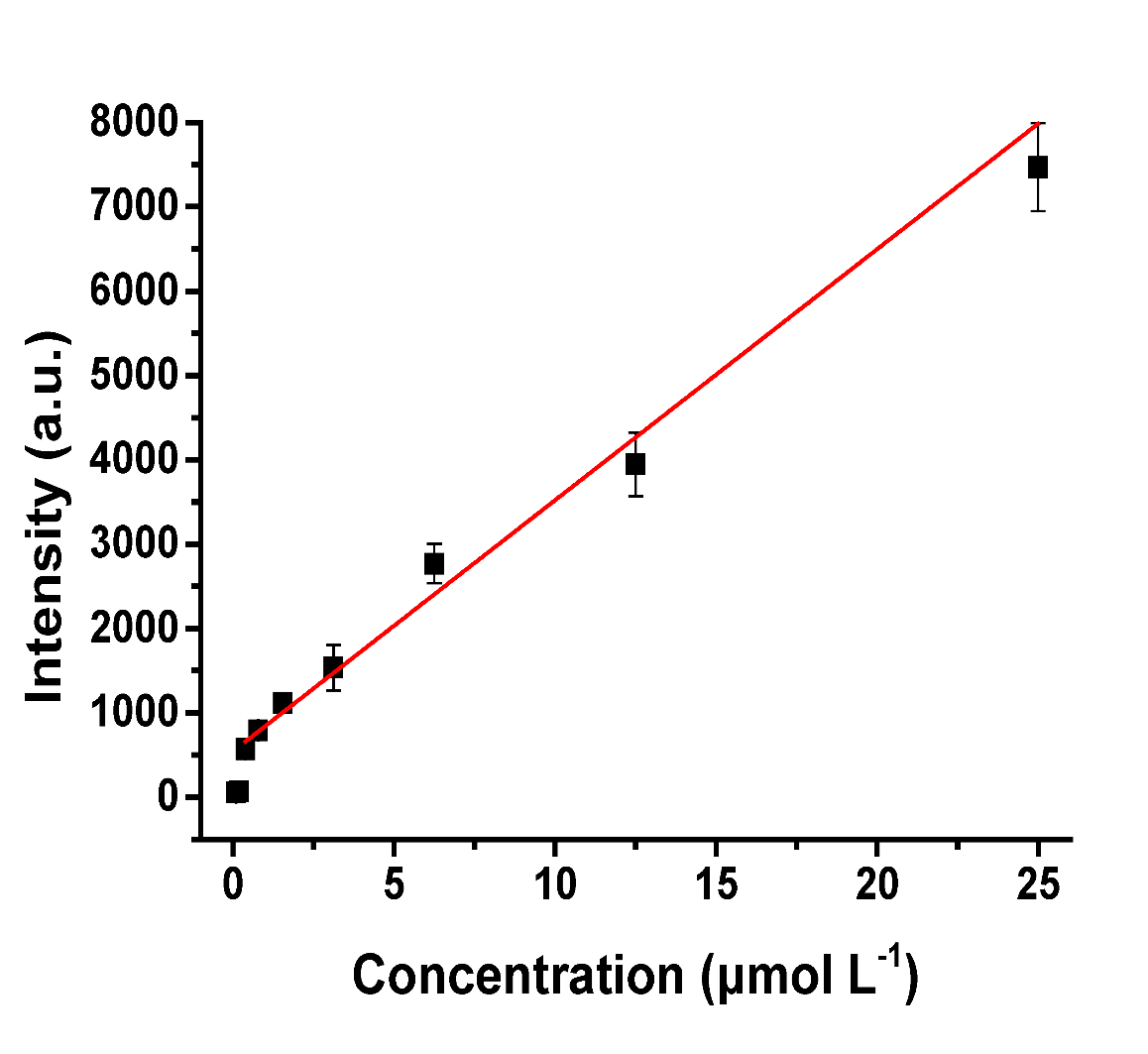


Supplementary Figure S2. Inverse correlations were observed between the LPC16:0 (D0) concentration and Lactate (D0) and Procalcitonin (D0) level. (A) LPC16:0 (D0) & lactate : r =-0.183 , *P =0.062 ,* (B) LPC16:0 (D0) & Procalcitonin : r =-0.196 , *P =0.047*


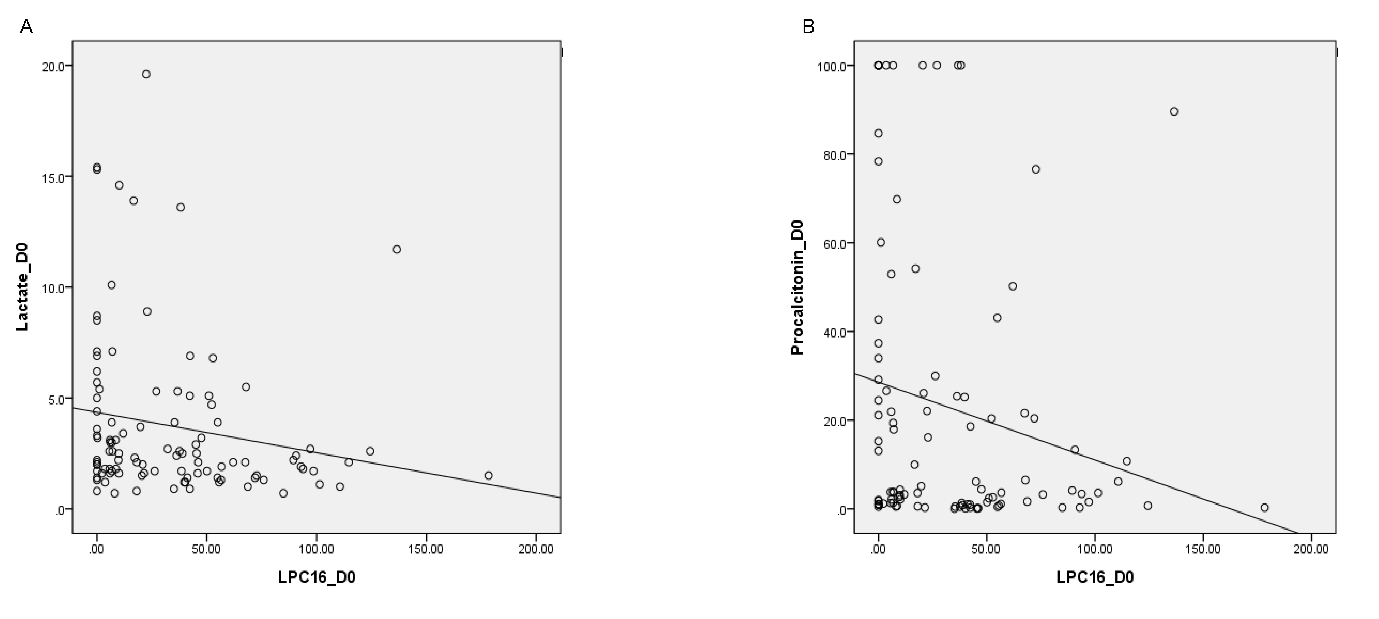


| Supplementary Figure S3. Life Table for LPC16:0 (D1-D1) >7.288 |
| --- |
| \| **Time Days** \| **Number at Risk (Nt)** \| **Number of Deaths (Dt)** \| **Survival Probability** \| \| --- \| --- \| --- \| --- \| \| 0 \| 57 \| 0 \| 1 \| \| 1 \| 57 \| 0 \| 1 \| \| 2 \| 57 \| 0 \| 1 \| \| 3 \| 57 \| 0 \| 1 \| \| 6 \| 57 \| 0 \| 1 \| \| 7 \| 57 \| 0 \| 1 \| \| 8 \| 57 \| 0 \| 1 \| \| 11 \| 57 \| 0 \| 1 \| \| 12 \| 57 \| 0 \| 1 \| \| 14 \| 57 \| 0 \| 1 \| \| 17 \| 57 \| 0 \| 1 \| \| 18 \| 56 \| 1 \| 0.982 \| \| 21 \| 55 \| 1 \| 0.965 \| \| 22 \| 55 \| 0 \| 0.965 \| \| 23 \| 54 \| 1 \| 0.947 \| \| 28 \| 53 \| 1 \| 0.93 \| |

Supplementary Figure S4. Life Table for LPC16:0 (D1-D1) ≤7.288

| **Time Days** | **Number at Risk (Nt)** | **Number of Deaths (Dt)** | **Survival Probability** |
| --- | --- | --- | --- |
| 0 | 55 | 1 | 0.982 |
| 1 | 54 | 1 | 0.964 |
| 2 | 53 | 1 | 0.946 |
| 3 | 47 | 6 | 0.839 |
| 6 | 43 | 4 | 0.768 |
| 7 | 42 | 1 | 0.750 |
| 8 | 41 | 1 | 0.732 |
| 11 | 40 | 1 | 0.714 |
| 12 | 39 | 1 | 0.696 |
| 14 | 38 | 1 | 0.679 |
| 17 | 37 | 1 | 0.661 |
| 18 | 37 | 0 | 0.661 |
| 21 | 37 | 0 | 0.661 |
| 22 | 36 | 1 | 0.643 |
| 23 | 36 | 0 | 0.643 |
| 28 | 36 | 0 | 0.643 |
